# Supplementary material for: Elastic–plastic fracture analysis of pressure pipelines with axial cracks based on the interaction integral method
Source: PLoS One. 2024 Dec 26;19(12):e0301015. doi: 10.1371/journal.pone.0301015 (PMC11670942; doi:10.1371/journal.pone.0301015)
Supplement: S3 File — (DOCX) [file pone.0301015.s004.docx]

/FILNAME,Pipeline failure analysis,0

/PREP7

ET,1,SHELL281 ! Define material units

! Setting material unit keywords

KEYOPT,1,1,0

KEYOPT,1,8,2

KEYOPT,1,9,0

! Define material parameters

MP,EX,1,2.06E11

MP,PRXY,1,0.3

TB,BISO,1,1,2,

TBTEMP,0

!TBDATA,,300E6,5E9,,,,

TBDATA,,560E6,0,,,,

! Define material cross-section parameters

sect,1,shell,,

secdata, 0.003,1,30,3

secdata, 0.003,1,30,3

secdata, 0.003,1,30,3

secdata, 0.003,1,30,3

secdata, 0.003,1,30,3

secdata, 0.0034,1,30,3

secoffset,MID

seccontrol,,,, , , ,

! modelling

CYLIND,0.61, ,0,4.6,0,180,

CYLIND,0.61, ,4.6,4.680,0,180,

VDELE, 1,2

ADELE, 2, , ,1

ADELE, 1, , ,1

ADELE, 4, , ,1

ADELE, 5, , ,1

ADELE, 6, , ,1

ADELE, 7, , ,1

ADELE, 9, , ,1

ADELE, 10,, ,1

NUMMRG,KP, , , ,LOW

FLST,2,2,5,ORDE,2

FITEM,2,3

FITEM,2,8

AGLUE,P51X

LPLOT

KSCON,4,0.001,1,10,0.75,

! Create a local coordinate system

LOCAL,11,1,0,0,0, , , ,1,1,

! meshing

ESIZE,0.08

ESYS, 11

AMESH,ALL

ESYS,0

! Access to the solution

/SOL

NLGEOM,1 ! Activation of large deformation analysis

!NSUBST,100,100,100

!OUTRES,ERASE

!OUTRES,ALL,ALL

!TIME,1

! Define displacement boundary conditions.

NSEL,ALL

NSEL,S,LOC,Z,0

NPLOT

D,ALL, , , , , ,UX,UY,UZ, , ,

ALLSEL,ALL

NSEL,ALL

NSEL,S,LOC,Z,4.680

NPLOT

DSYM,SYMM,Z, ,

ALLSEL,ALL

!D,ALL, , , , , ,UX,UY,UZ, , ,

!ALLSEL,ALL

FLST,5,3,4,ORDE,3

FITEM,5,7

FITEM,5,-8

FITEM,5,16

LSEL,S, , ,P51X

LPLOT

NSLL,R,1

NPLOT

DSYM,SYMM,Y, ,

ALLSEL,ALL

! Defining pressure loads on pipelines

SFA,3,1,PRES,8E6

SFA,8,1,PRES,8E6

OUTRES,ALL,ALL,

TIME,1

AUTOTS,1

NSUBST,100,100 , 100,1

KBC,0

! Start solving

SOLVE

FINISH

/POST26

FILE,'Pipelinefailureanalysis','rst','.'

/UI,COLL,1

NUMVAR,200

SOLU,191,NCMIT

STORE,MERGE

FILLDATA,191,,,,1,1

REALVAR,191,191

FORCE,TOTAL

ANSOL,2,166,EPPL,EQV,EPPLEQV_2

NUMVAR,200

FILLDATA,191,,,,1,1

REALVAR,191,191

XVAR,1

PLVAR,2,
