# Supplementary material for: Elastic–plastic fracture analysis of pressure pipelines with axial cracks based on the interaction integral method
Source: PLoS One. 2024 Dec 26;19(12):e0301015. doi: 10.1371/journal.pone.0301015 (PMC11670942; doi:10.1371/journal.pone.0301015)
Supplement: S4 File — (DOCX) [file pone.0301015.s005.docx]

/FILNAME,Pipeline failure analysis,0

/PREP7

ET,1,SHELL281 ! Define material units

! Setting material unit keywords.

KEYOPT,1,1,0

KEYOPT,1,8,2

KEYOPT,1,9,0

! Define material parameters

MP,EX,1,2.03E11

MP,PRXY,1,0.25

TB,BISO,1,1,2,

TBTEMP,0

!TBDATA,,300E6,5E9,,,,

TBDATA,,560E6,0,,,,

! Define material cross-section parameters

sect,1,shell,,

secdata, 0.003,1,30,3

secdata, 0.003,1,30,3

secdata, 0.003,1,30,3

secdata, 0.003,1,30,3

secdata, 0.003,1,30,3

secdata, 0.003,1,30,3

secoffset,MID

seccontrol,,,, , , ,

! modelling

CYLIND,0.61, ,0,4.6,0,180,

CYLIND,0.61, ,4.6,4.615,0,180,

VDELE, 1,2

ADELE, 2, , ,1

ADELE, 1, , ,1

ADELE, 4, , ,1

ADELE, 5, , ,1

ADELE, 6, , ,1

ADELE, 7, , ,1

ADELE, 9, , ,1

ADELE, 10,, ,1

NUMMRG,KP, , , ,LOW

FLST,2,2,5,ORDE,2

FITEM,2,3

FITEM,2,8

AGLUE,P51X

LPLOT

KSCON,4,0.001,1,10,0.75,

! Create a local coordinate system.

LOCAL,11,1,0,0,0, , , ,1,1,

! meshing

ESIZE,0.08

ESYS, 11

AMESH,ALL

ESYS,0

/VIEW,1,1,1,1

/ANG,1

/REP,FAST
