# Supplementary material for: Elastic–plastic fracture analysis of pressure pipelines with axial cracks based on the interaction integral method
Source: PLoS One. 2024 Dec 26;19(12):e0301015. doi: 10.1371/journal.pone.0301015 (PMC11670942; doi:10.1371/journal.pone.0301015)
Supplement: Attachment — Submitted filename: Response to Reviewers.docx [file pone.0301015.s006.docx]

# List of responses

**Title：Elastic–plastic fracture analysis of pressure pipelines with axial cracks based on the interaction integral method**

**Manuscript** **ID：PONE-D-23-24376**

Dear Editor and Reviewers,

On behalf of my co-authors, we are very grateful to you for giving us an opportunity to revise our manuscript. we appreciate you very much for your positive and constructive comments and suggestions on our manuscript. We have studied reviewers' comments carefully and tried our best to revise our manuscript according to the comments. The following are the responses and revisions I have made in response to the reviewers' questions and suggestions on an item-by-item basis. In this revised version, changes to our manuscript were all highlighted within the document by using **red-colored text**. Thanks again to the hard work of the editor and reviewer!

Response to the referee's comments.

## ACADEMIC EDITOR:

- I just received comments from one reviewer.
- Both English and presentations should be significantly improved. The novelty of this manuscript should be clearly stated through literature review.
- Refertences with last three years should be updated to help you well define your research problem.

We also appreciate your clear and detailed feedback and hope that the explanation has fully addressed all of your concerns. In the remainder of this letter, we discuss each of your comments individually along with our corresponding responses.

1. **Both English and presentations should be significantly improved. The novelty of this manuscript should be clearly stated through literature review.**

Reply: Thank you very much for this important comment.

Firstly, the English and presentations of Abstract, Introduction, Materials and Methods, Results and Analysis, and Conclusion have been revised or rewritten. Please see the 'Revised Manuscript with Track Changes'.

Secondly, the content “

The novelty of this manuscript is mainly as follows:

(1) The interaction integral technique is utilised to compute the stress intensity factor of pipeline fissures, thereby circumventing the reliance of conventional numerical fracture analysis on meshes, which in turn enhances the precision and efficiency of the calculations.

(2) The text presents the Ramberg-Osgood model for pipeline steel material, analyzing the impact of minor plastic deformation near pipeline crack tips on the stress intensity factor, based on the elastic fracture mechanics of the Irwin line. This improves the dependability of pipeline safety analyses.

” has been added(Please see the revised manuscript at the fourth paragraph of page 2)

2. **Refertences with last three years should be updated to help you well define your research problem.**

Reply: Thank you very much for this important comment.

Text coloured in red are references that have been replaced, the reference “

1. Xie M, Wang Y, Xiong W, Zhao J, Pei X.A crack propagation method for pipelines with interacting corrosion and crack defects. Sensors. 2022;22:986. https://doi.org/ 10.3390/s22030986.

2. Wu T, Yan M, Yu L, Zhao H, Sun C, Yin F, et al. Stress corrosion of pipeline steel under disbonded coating in a SRB-containing environment. Corros Sci. 2019;157:518-530.https://doi.org/10.1016/j.corsci.2019.06.026.

3. Dong S, Zhang L, Zhang H.Crack propagation rate of hydrogen-induced cracking in high sulfur-containing pipelines. Eng. Fail. Anal. 2021;123:105271. [https://doi.org/10.1016/j.en-gfailanal.2021.105271.](https://doi.org/10.1016/j.engfailanal.2021.105271.)

4. Yin T, Wang J, Zhao H,Zhou L, Xue Z, Wang H.Research on Filling Strategy of Pipeline Multi-Layer Welding for Compound Narrow Gap Groove. Materials. 2022;15(17): 5967. https://doi.org/10.3390/ma15175967.

5. Liu Q, Li N, Shen ZX, Zhao M, Xie J, Zhu G, et al. Calculation model and experimental study of the collapse strength of titanium alloy tubing and casing. Scientific Reports. 2022 Mar 16;12(1). https://doi.org/10.1038/s41598-022-08636-9.

6. Mária Hagarová, Gabriela Baranová, Pavel Peterka, Branislav Buľko, Martina Hrubovčáková, Vladimír Girman. Failure analysis of a gas pipeline at the kinked dent location with crack indications. Eng. Fail. Anal. 2023;153:107579. <https://doi.org/10.1016/j.engfailanal.2023.107579.>

7. Zhen Y, Liu Y, Cao Y, Niu R. Study on crack tip opening angle evolutions during dyna-mic crack propagation of pipelines. Fatigue Fract Eng Mater Struct.2023;46(5):1969-1978.https://doi.org/10.1111/ffe.13976.

8. Sun C, Wang Q, Li Y, Li Y, Liu Y. Numerical simulation and analytical prediction of resi-dual strength for elbow pipes with erosion defects. Materials. 2022;15(21):7479. https://doi.org/10.3390/ma15217479.

9. Zhang B, Wan W.A transient-features-based diagnostic method of multi incipient cracks in pipeline systems. Int. J. Press. Vessel. Pip. 2022;199:104701. https://doi.org/10.1016/j.ijpvp.2022.104701.

10. Wang Y, Zhang P, Qin G. Reliability assessment of pitting corrosion of pipeline under spatiotemporal earthquake including spatial-dependent corrosion growth. Process Saf. Environ. 2021;148:166–78. https://doi.org/10.1016/j.psep.2020.10.005.

11. H. C. Andrade, J. Trevelyan, E. D. Leone. Direct evaluation of stress intensity factors and T-stress for bimaterial interface cracks using the extended isogeometric boundary element method. Theor Appl Fract Mech. 2023;127:104091. https://doi.org/10.1016/j.taf-mec.2023.104091.

12. Zhao L, Shi Z, Wang Z, Yang F. An Investigation of a New Parameter Based on the Plastic Strain Gradient to Characterize Composite Constraint around the Crack Front at a Low Temperature. Materials. 2022;15(3):881. https://doi.org/10.3390/ma15030881.

13. Abdellah MY, Alfattani R, Alnaser IA, Abdel-Jaber GT. Stress Distribution and Fracture Toughness of Underground Reinforced Plastic Pipe Composite. Polymers. 2021 Jun 30;13(13):2194. https://doi.org/10.3390/polym13132194.

14. Ince A, Glinka G. Approximation modeling framework for elastic-plastic stress-strain fiel-ds near cracks with a small finite crack tip radius. Theoretical and Applied Fracture Me-chanics. 2022 Oct;121:103452. https://doi.org/10.1016/j.tafmec.2022.103452.

15. M. Graba. Numerical Evaluation of the Fracture Process of 41Cr4 Steel: Analysis of Cracks Grown in a Plane Strain State Domination Based on Experimental Results. Materials. 2022 Oct 20;15(20):7361–1. https://doi.org/10.3390/ma15207361.

16. Ying Z, Zhanghua L, Chenxin W, Brice NF. Research on damage progression of drill string material based on the extended finite element method. Science Progress. 2021;104(3). doi:10.1177/00368504211042258.

17.N V Nguyena, D Leea, H Nguyen-Xuan, J Lee. A polygonal finite element approach for fatigue crack growth analysis of interfacial cracks. Theor Appl Fract Mech. 2020;108:102576. https://doi.org/10.1016/j.tafmec.2020.102576.

18.M T Mohammadi Anaei, A Khosravifard, T Q Bui. Analysis of fracture mechanics and fatigue crack growth in moderately thick plates using an efficient meshfree approach. Theor. Appl. Fract. Mech. 2021;113:102943. https://doi.org/10.1016/j.tafmec.2021.102943.

19.Zhu X, P Lam, Chao Y. Constraint-dependent CTOA determination for stable ductile cra-ck growth. Eng Fract Mech.2022;271:108651. https://doi.org/10.1016/j.engfracmech.2022.108651.

20.Reza Jamshidi Chenari, Azadeh Azadegan. Dynamic elasto-plastic finite element analysis of buried pipelines in slopes. Electron J Geotech Eng. 2016; (21.13): 4797-4811.

21.S Kei , H Tei , M Ninshu , M Hidekazu. Characteristic-tensor method for efficient esti-mation of stress-intensity factors of three-dimensional cracks. Eng Fract Mech. 2021;257:10861. https://doi.org/10.1016/j.engfracmech.2021.108016.

22.Ye X, Cui S, Liu T, Ma Q, Liu G, Huang Z, et al. Microstructure Characterization and Strengthening Mechanism Analysis of X100 Pipeline Steel. Coatings. 2023; 13(4):706. <https://doi.org/10.3390/coatings13040706.>

23.Shoheib M. A novel correlation models for predicting stress intensity factor of semi-elliptical crack in welded pipelines under cyclic internal pressure based on Bézier base XI-GA. Int. J. Press. Vessel. Pip. 2023;206:105052. https://doi.org/10.1016/j.ijpvp.2023.105052.

24. Hou S, Cai Z, Zhu Y, Zhao Q, Chen Y, Gao H, et al. Elasto-Plastic Fracture Mechanics Analysis of the Effect of Shot Peening on 300M Steel. Materials. 2021;14(13):3538. <https://doi.org/10.3390/ma14133538>.

25. Li L, Deng You-sheng. Fracture analysis-based mode-I stress intensity factors of crack under fracture grouting in elastic–plastic soils. Scientific Reports. 2023 Jan 25;13(1). https://doi.org/10.1038/s41598-023-28649-2.

26. Bose S, Li S, Mele E, Silberschmidt VV. Fracture behaviour and toughening mechanisms of dry and wet collagen. Acta Biomaterialia. 2022 Apr 1;142:174–84. https://doi.org/10.1016/j.actbio.2022.02.001.

27. Zhang Z, Liu R, Zhang K, Sun D, Zhang Y, et al. Jk-integral applied to mixed-mode fa-tigue crack propagation and life prediction in metal welding interface. Int. J. Solids Str-uct. 2023;268:112184. https://doi.org/10.1016/j.ijsolstr.2023.112184.

28. Kowalczyk, J. Modeling the Depth of Surface Cracks in Brake Disc. Materials 2021, 14, 3890.

~~29. Chao, Y.J.; Zhu, X.K.; Kim, Y.; Lar, P.S.; Pechersky, M.J.; Morgan, M.J. Characterization of crack-tip field and constraint for bending specimens under large-scale yielding. International Journal of Fracture 2004, 127, 283-302.~~

29. Guo, J.; Hui, C.Y.; Liu, M.; Zehnder, A.T. The stress field near the tip of a plane stress crack in a gel consisting of chemical and physical cross-links. Proceedings of The Royal Society A Mathematical Physical and Engineering Sciences 2019, 475, 20180863.

30. Karimi, R. A patient-specific numerical modeling of the spontaneous coronary artery dissection in relation to atherosclerosis. Computer Methods and Programs in Biomedicine: An International Journal Devoted to the Development, Implementation and Exchange of Computing Methodology and Software Systems in Biomedical Research and Medical Practice 2019, 182, 105060.

” has been added(Please see the revised manuscript at the references of page 12-13).

We would like to take this opportunity to thank you for all your time involved and this great opportunity for us to improve the manuscript. We hope you will find this revised version satisfactory.

Sincerely,

Qi Song, Huifen Peng

## Reviewer: #1

A review of the full paper found that acceptance in its current form for publication in the journal of PLOS ONE is not recommended. The paper is poor-written. Reconsider after addressing the following problems:

1. English style is the primary problem. Currently, I cannot understand this study clearly unless a native speaker to re-check the grammar and typos.

2. There are too much old literature cited in the reference list, should replace which with some recent literatures about soil research and application of soil uncertainties. Authors can consult: https://doi.org/10.1016/j.tws.2020.107404;

https://doi.org/10.1016/j.psep.2020.10.005;

https://doi.org/10.3390/s22030986.

3.Discussions need to increase the broad applicability of the model. I cannot capture the novelty of this work.

4.Conclusion part is poor written, the core contributions are required to list.

We appreciate your clear and detailed feedback and hope that the explanation has fully addressed all of your concerns. In the remainder of this letter, we discuss each of your comments individually along with our corresponding responses.

1. **English style is the primary problem. Currently, I cannot understand this study clearly unless a native speaker to re-check the grammar and typos.**

Reply: Thank you very much for this important comment.

The English and presentations of Abstract, Introduction, Materials and Methods, Results and Analysis, and Conclusion have been revised or rewritten. Please see the 'Revised Manuscript with Track Changes'.

2. **There are too much old literature cited in the reference list, should replace which with some recent literatures about soil research and application of soil uncertainties. Authors can consult: https://doi.org/10.1016/j.tws.2020.107404; https://doi.org/10.1016/j.psep.2020.10.005;** **https://doi.org/10.3390/s22030986.**

Reply: Thank you very much for this important comment.

Text coloured in red are references that have been replaced, two recommended references in the issue are cited, with the following serial numbers: No.1 and No.10. The reference “

1. Xie M, Wang Y, Xiong W, Zhao J, Pei X.A crack propagation method for pipelines with interacting corrosion and crack defects. Sensors. 2022;22:986. https://doi.org/ 10.3390/s22030986.

2. Wu T, Yan M, Yu L, Zhao H, Sun C, Yin F, et al. Stress corrosion of pipeline steel under disbonded coating in a SRB-containing environment. Corros Sci. 2019;157:518-530.https://doi.org/10.1016/j.corsci.2019.06.026.

3. Dong S, Zhang L, Zhang H.Crack propagation rate of hydrogen-induced cracking in high sulfur-containing pipelines. Eng. Fail. Anal. 2021;123:105271. [https://doi.org/10.1016/j.en-gfailanal.2021.105271.](https://doi.org/10.1016/j.engfailanal.2021.105271.)

4. Yin T, Wang J, Zhao H,Zhou L, Xue Z, Wang H.Research on Filling Strategy of Pipeline Multi-Layer Welding for Compound Narrow Gap Groove. Materials. 2022;15(17): 5967. https://doi.org/10.3390/ma15175967.

5. Liu Q, Li N, Shen ZX, Zhao M, Xie J, Zhu G, et al. Calculation model and experimental study of the collapse strength of titanium alloy tubing and casing. Scientific Reports. 2022 Mar 16;12(1). https://doi.org/10.1038/s41598-022-08636-9.

6. Mária Hagarová, Gabriela Baranová, Pavel Peterka, Branislav Buľko, Martina Hrubovčáková, Vladimír Girman. Failure analysis of a gas pipeline at the kinked dent location with crack indications. Eng. Fail. Anal. 2023;153:107579. <https://doi.org/10.1016/j.engfailanal.2023.107579.>

7. Zhen Y, Liu Y, Cao Y, Niu R. Study on crack tip opening angle evolutions during dyna-mic crack propagation of pipelines. Fatigue Fract Eng Mater Struct.2023;46(5):1969-1978.https://doi.org/10.1111/ffe.13976.

8. Sun C, Wang Q, Li Y, Li Y, Liu Y. Numerical simulation and analytical prediction of resi-dual strength for elbow pipes with erosion defects. Materials. 2022;15(21):7479. https://doi.org/10.3390/ma15217479.

9. Zhang B, Wan W.A transient-features-based diagnostic method of multi incipient cracks in pipeline systems. Int. J. Press. Vessel. Pip. 2022;199:104701. https://doi.org/10.1016/j.ijpvp.2022.104701.

10. Wang Y, Zhang P, Qin G. Reliability assessment of pitting corrosion of pipeline under spatiotemporal earthquake including spatial-dependent corrosion growth. Process Saf. Environ. 2021;148:166–78. https://doi.org/10.1016/j.psep.2020.10.005.

11. H. C. Andrade, J. Trevelyan, E. D. Leone. Direct evaluation of stress intensity factors and T-stress for bimaterial interface cracks using the extended isogeometric boundary element method. Theor Appl Fract Mech. 2023;127:104091. https://doi.org/10.1016/j.taf-mec.2023.104091.

12. Zhao L, Shi Z, Wang Z, Yang F. An Investigation of a New Parameter Based on the Plastic Strain Gradient to Characterize Composite Constraint around the Crack Front at a Low Temperature. Materials. 2022;15(3):881. https://doi.org/10.3390/ma15030881.

13. Abdellah MY, Alfattani R, Alnaser IA, Abdel-Jaber GT. Stress Distribution and Fracture Toughness of Underground Reinforced Plastic Pipe Composite. Polymers. 2021 Jun 30;13(13):2194. https://doi.org/10.3390/polym13132194.

14. Ince A, Glinka G. Approximation modeling framework for elastic-plastic stress-strain fiel-ds near cracks with a small finite crack tip radius. Theoretical and Applied Fracture Me-chanics. 2022 Oct;121:103452. https://doi.org/10.1016/j.tafmec.2022.103452.

15. M. Graba. Numerical Evaluation of the Fracture Process of 41Cr4 Steel: Analysis of Cracks Grown in a Plane Strain State Domination Based on Experimental Results. Materials. 2022 Oct 20;15(20):7361–1. https://doi.org/10.3390/ma15207361.

16. Ying Z, Zhanghua L, Chenxin W, Brice NF. Research on damage progression of drill string material based on the extended finite element method. Science Progress. 2021;104(3). doi:10.1177/00368504211042258.

17.N V Nguyena, D Leea, H Nguyen-Xuan, J Lee. A polygonal finite element approach for fatigue crack growth analysis of interfacial cracks. Theor Appl Fract Mech. 2020;108:102576. https://doi.org/10.1016/j.tafmec.2020.102576.

18.M T Mohammadi Anaei, A Khosravifard, T Q Bui. Analysis of fracture mechanics and fatigue crack growth in moderately thick plates using an efficient meshfree approach. Theor. Appl. Fract. Mech. 2021;113:102943.https://doi.org/10.1016/j.tafmec.2021.102943.

19.Zhu X, P Lam, Chao Y. Constraint-dependent CTOA determination for stable ductile cra-ck growth. Eng Fract Mech.2022;271:108651. https://doi.org/10.1016/j.engfracmech.2022.108651.

20.Reza Jamshidi Chenari, Azadeh Azadegan. Dynamic elasto-plastic finite element analysis of buried pipelines in slopes. Electron J Geotech Eng. 2016; (21.13): 4797-4811.

21.S Kei , H Tei , M Ninshu , M Hidekazu. Characteristic-tensor method for efficient esti-mation of stress-intensity factors of three-dimensional cracks. Eng Fract Mech. 2021;257:10861. https://doi.org/10.1016/j.engfracmech.2021.108016.

22.Ye X, Cui S, Liu T, Ma Q, Liu G, Huang Z, et al. Microstructure Characterization and Strengthening Mechanism Analysis of X100 Pipeline Steel. Coatings. 2023; 13(4):706. <https://doi.org/10.3390/coatings13040706.>

23.Shoheib M. A novel correlation models for predicting stress intensity factor of semi-elliptical crack in welded pipelines under cyclic internal pressure based on Bézier base XI-GA. Int. J. Press. Vessel. Pip. 2023;206:105052. https://doi.org/10.1016/j.ijpvp.2023.105052.

24. Hou S, Cai Z, Zhu Y, Zhao Q, Chen Y, Gao H, et al. Elasto-Plastic Fracture Mechanics Analysis of the Effect of Shot Peening on 300M Steel. Materials. 2021;14(13):3538. <https://doi.org/10.3390/ma14133538>.

25. Li L, Deng You-sheng. Fracture analysis-based mode-I stress intensity factors of crack under fracture grouting in elastic–plastic soils. Scientific Reports. 2023 Jan 25;13(1). https://doi.org/10.1038/s41598-023-28649-2.

26. Bose S, Li S, Mele E, Silberschmidt VV. Fracture behaviour and toughening mechanisms of dry and wet collagen. Acta Biomaterialia. 2022 Apr 1;142:174–84. https://doi.org/10.1016/j.actbio.2022.02.001.

27. Zhang Z, Liu R, Zhang K, Sun D, Zhang Y, et al. Jk-integral applied to mixed-mode fa-tigue crack propagation and life prediction in metal welding interface. Int. J. Solids Str-uct. 2023;268:112184. https://doi.org/10.1016/j.ijsolstr.2023.112184.

28. Kowalczyk, J. Modeling the Depth of Surface Cracks in Brake Disc. Materials 2021, 14, 3890.

~~29. Chao, Y.J.; Zhu, X.K.; Kim, Y.; Lar, P.S.; Pechersky, M.J.; Morgan, M.J. Characterization of crack-tip field and constraint for bending specimens under large-scale yielding. International Journal of Fracture 2004, 127, 283-302.~~

29. Guo, J.; Hui, C.Y.; Liu, M.; Zehnder, A.T. The stress field near the tip of a plane stress crack in a gel consisting of chemical and physical cross-links. Proceedings of The Royal Society A Mathematical Physical and Engineering Sciences 2019, 475, 20180863.

30. Karimi, R. A patient-specific numerical modeling of the spontaneous coronary artery dissection in relation to atherosclerosis. Computer Methods and Programs in Biomedicine: An International Journal Devoted to the Development, Implementation and Exchange of Computing Methodology and Software Systems in Biomedical Research and Medical Practice 2019, 182, 105060.

” has been added(Please see the revised manuscript at the references of page 12-13).

3. **Discussions need to increase the broad applicability of the model. I cannot capture the novelty of this work.**

Reply: Thank you very much for this important comment.

**3.1 With regard to discussions need to increase the broad applicability of the model**

I appreciate your concern regarding the broad applicability and novelty of our research. I believe there are several unique aspects of our study that enhance its potential for wider applicability.

First and foremost, our research employs an innovative approach to tackle specific issues, which can address challenges that existing methods struggle with. This novel approach holds promise for applications across multiple domains, thus expanding its scope of relevance.

Secondly, Our work can be seen as an initial endeavor, upon which future research can build and refine, thereby enhancing its broader applicability.

Lastly, our research also offers a method and framework that can be customized and adjusted according to specific needs, making it adaptable to diverse application scenarios. This flexibility allows our approach to be more readily employed in various fields and problem-solving contexts.

In summary, although our work may not comprehensively cover all situations at present, it possesses uniqueness and adaptability, rendering it a promising foundation for future research endeavors and thereby enhancing its wider applicability.

The content “

In summary, the method proposed in this paper can effectively support pipeline safety assessments, particularly when the ratio of initial crack size to the plastic zone size at the crack tip complies with conditions of limited yielding. To further enhance the comprehensiveness of this research field, future studies could expand their scope to encompass fracture analysis in pipelines with extensive yielding at the crack tip, thereby establishing more efficient models that consider both large-scale yielding at the crack tip and the incorporation of the Dugdale model.

” has been added(Please see the revised manuscript at the third paragraph of page 10)

**3.2 With regard to novelty of this work**

The content “

The novelty of this manuscript is mainly as follows:

(1) The interaction integral technique is utilised to compute the stress intensity factor of pipeline fissures, thereby circumventing the reliance of conventional numerical fracture analysis on meshes, which in turn enhances the precision and efficiency of the calculations.

(2) The text presents the Ramberg-Osgood model for pipeline steel material, analyzing the impact of minor plastic deformation near pipeline crack tips on the stress intensity factor, based on the elastic fracture mechanics of the Irwin line. This improves the dependability of pipeline safety analyses.

” has been added(Please see the revised manuscript at the fourth paragraph of page 2)

4. **Conclusion part is poor written, the core contributions are required to list.**

Reply: Thank you very much for this important comment.

The content “

**This paper introduces a three-dimensional finite element model for conducting elastoplastic fracture analysis in pipelines containing axial cracks. We propose a novel fracture analysis method based on the Ramberg-Osgood material model and the Mises yield criterion, which accounts for the plastic zone effect at the crack tip. This method takes into consideration the initial crack length, pipeline geometric parameters, and internal pressure.**

**Our approach provides accurate calculations of the stress intensity factor and is capable of predicting the pipeline's elastic and plastic limit pressures. To quantify the influence of the plastic zone at the crack tip on the pipeline's stress intensity factor, we employ the relative error before and after the plastic correction of the stress intensity factor. The effectiveness of the proposed method is demonstrated through experimental results.**

**Several key findings are listed below:**

(1) **The SIF increases as the pipeline radius increases, and it decreases as the pipeline thickness increases.**
(2) **The elastic and plastic limit load of the pipeline decreases as the initial crack length increases, and the time required for the initial yield decreases gradually as the initial crack length increases.**
(3) **The change rule of the plastic zone at the crack tip with pipeline relative crack length is nonlinear. When the pipeline relative crack length is smaller than 0.007, the nonlinear change is not obvious, when the pipeline relative crack length reached 0.01 , plastic zone at the crack tip increases sharply as the relative crack length increase, which caused a further decrease in Elastic constraint of pipeline, indicating that the plastic zone at the crack tip expanded unlimitedly, which leads to the failure of the pipeline.**
(4) **The relative error of the SIF before and after plasticity correction overall increased as the internal pressure of pipeline increases. the relative error increased relatively slowly when the internal pressure was in the range of 1 MPa and 4 MPa and then increased quickly when the internal pressure was biggler than 6 MPa, the highest relative error is 23.63% when the internal pressure was equal to 10 MPa, which means it is necessary to consider the effect of crack tip plasticity on the SIF When the internal pressure of the pipeline is higher than 8 MPa.**

**In summary, the method proposed in this paper can effectively support pipeline safety assessments, particularly when the ratio of initial crack size to the plastic zone size at the crack tip complies with conditions of limited yielding. To further enhance the comprehensiveness of this research field, future studies could expand their scope to encompass fracture analysis in pipelines with extensive yielding at the crack tip, thereby establishing more efficient models that consider both large-scale yielding at the crack tip and the incorporation of the Dugdale model.**

” has been added(Please see the revised manuscript at the second paragraph of page 11)

We would like to take this opportunity to thank you for all your time involved and this great opportunity for us to improve the manuscript. We hope you will find this revised version satisfactory.

Sincerely,

Qi Song, Huifen Peng

Funding Statement: This research was funded by National Natural Science Foundation of China, grant number “51674088”.
