# Supplementary material for: Elastic–plastic fracture analysis of pressure pipelines with axial cracks based on the interaction integral method
Source: PLoS One. 2024 Dec 26;19(12):e0301015. doi: 10.1371/journal.pone.0301015 (PMC11670942; doi:10.1371/journal.pone.0301015)
Supplement: Attachment — Submitted filename: Response to Reviewers.docx [file pone.0301015.s007.docx]

# List of responses

**Title：Elastic–plastic fracture analysis of pressure pipelines with axial cracks based on the interaction integral method**

**Manuscript** **ID：PONE-D-23-24376R1**

Dear Editor and Reviewers,

On behalf of my co-authors, we are very grateful to you for giving us an opportunity to revise our manuscript. we appreciate you very much for your positive and constructive comments and suggestions on our manuscript. We have studied reviewers' comments carefully and tried our best to revise our manuscript according to the comments. The following are the responses and revisions I have made in response to the reviewers' questions and suggestions on an item-by-item basis. In this revised version, changes to our manuscript were all highlighted within the document by using **red-colored text**. Thanks again to the hard work of the editor and reviewer!

Response to the referee's comments.

## Reviewer: #1

1. It is not yet evident in detail how the selection of the boundary condition for the constraint of the fixed end of the right end face of the pipeline is based on the actual physical conditions of the pressure pipeline. Suggest providing a reasonable explanation.

2. The article did not analyze the sensitivity of the grid, which may cause some confusion. Although the interaction integration method can improve the accuracy and efficiency of calculating fracture parameters and reduce the requirements for grids, it does not mean that the influence of grids does not need to be considered entirely. Therefore, it is recommended that relevant explanations be provided for the lack of grid sensitivity analysis.

3. It is recommended to provide clearer crack modeling in the finite element model diagram so that readers can understand the model more clearly. This may include more detailed crack geometry in the model.

We appreciate your clear and detailed feedback and hope that the explanation has fully addressed all of your concerns. In the remainder of this letter, we discuss each of your comments individually along with our corresponding responses.

1. **It is not yet evident in detail how the selection of the boundary condition for the constraint of the fixed end of the right end face of the pipeline is based on the actual physical conditions of the pressure pipeline. Suggest providing a reasonable explanation.**

Reply: Thank you very much for this important comment.

The original drawing was wrong, and the boundary conditions on the right end face of the 1/4 computational model were symmetrically constrained. Fig 5

has been modified accordingly.(Please see the revised manuscript at the first paragraph of page 7)

**Fig 5.** (a) Finite element model of pipeline with axial cracks and (b) Pipeline restraint method

2. **The article did not analyze the sensitivity of the grid, which may cause some confusion. Although the interaction integration method can improve the accuracy and efficiency of calculating fracture parameters and reduce the requirements for grids, it does not mean that the influence of grids does not need to be considered entirely. Therefore, it is recommended that relevant explanations be provided for the lack of grid sensitivity analysis.**

Reply: Thank you very much for this important comment.

The content “

# 2.4 Grid sensitivity analysis

In order to analyze the sensitivity of the grid, setting node positions as shown in Fig 3, the results of the numerical analysis of the four nodes of the cracked tip with different mesh accuracies were analysed, as shown in Table 1, where the great relative error is defined as the relative error between the result of the calculation of the maximum number of cells and the number of nodes and the result of the calculation of the minimum number of cells and the number of nodes.

**Fig 3.** Node number corresponding location map

**Table 1.** Grid sensitivity analysis

| Node number | VonMises stress/MPa | | | | | | Large relative error / % |
| --- | --- | --- | --- | --- | --- | --- | --- |
|  | Number of units | 2227 | 2369 | 3453 | 4081 | 4786 |  |
|  | Number of nodes | 6680 | 7072 | 10384 | 12260 | 14367 |  |
| Node 1 |  | 229.59 | 235.59 | 242.96 | 246.89 | 247.52 | 7.81 |
| Node 2 |  | 228.34 | 234.92 | 238.06 | 242.56 | 245.19 | 7.37 |
| Node 3 |  | 227.79 | 231.85 | 235.06 | 238.66 | 241.45 | 5.10 |
| Node 4 |  | 226.62 | 230.77 | 234.38 | 237.92 | 239.12 | 5.51 |

From the analysis results, it can be seen that the node VonMises stress near the crack tip of the pipeline gradually decreases with the increase of the distance from the crack tip, the same node with the increase of the number of cells and nodes, the error of the numerical calculation results of the VonMises stress gradually decreases, and the relative error of the calculation results of different nodes with the minimum number of cells and nodes and the calculation results of the grid accuracy with the maximum number of cells and nodes is maximum 7.81% and minimum 5.10%, and it can be seen that the grid calculation accuracy of this paper meets the general accuracy requirements of the project. The relative error between the calculation results of different nodes with minimum number of cells and nodes and the calculation results with maximum number of cells and nodes is 7.81%, and the minimum is 5.10%, which can be seen from the above analysis.

” has been added(Please see the revised manuscript at the fourth paragraph of page 5-6)

3. **It is recommended to provide clearer crack modeling in the finite element model diagram so that readers can understand the model more clearly. This may include more detailed crack geometry in the model.**

Reply: Thank you very much for this important comment.

**With regard to provide clearer crack modeling in the finite element model, Fig 4**

**Fig 4.** Axial penetration cracks in pressure pipelines

” has been added(Please see the revised manuscript at the fifth paragraph of page 6)

We would like to take this opportunity to thank you for all your time involved and this great opportunity for us to improve the manuscript. We hope you will find this revised version satisfactory.

Sincerely,

Qi Song, Huifen Peng
